# The Tolman–Ehrenfest criterion of thermal equilibrium in scalar–tensor gravity

**DOI:** 10.1140/epjc/s10052-024-13632-6

**Published:** 2024-12-02

**Authors:** Numa Karolinski, Valerio Faraoni

**Affiliations:** 1 Physics Department, McGill University, 3600 Rue University, Montreal, QC H3A 2T8 Canada; 2 Department of Physics and Astronomy, Bishop’s University, 2600 College Street, Sherbrooke, QC J1M 1Z7 Canada

## Abstract

The Tolman–Ehrenfest criterion for the thermal equilibrium of a fluid at rest in a static general-relativistic geometry is generalized to scalar–tensor gravity. Surprisingly, the gravitational scalar field, which fixes the strength of the effective gravitational coupling, does not play a role in determining thermal equilibrium. As a result, heat does not sink more in a gravitational field where gravity is stronger.

## Introduction

Thermal physics in curved spacetime is rather intriguing. In the early days of general relativity (GR) Tolman, Ehrenfest [1–3], and then Eckart [4, 5] discussed the thermal equilibrium of fluids and heat conduction in the relativistic context. The concept of thermal equilibrium is not as general, or useful, in GR as it is in pre-relativistic physics, or even in special relativity. In fact, when spacetime becomes curved and dynamical, a thermal system (e.g., a fluid), is shaken around by the time-varying geometry described by the spacetime metric $g_{ab}$, spatial temperature gradients and fluid acceleration arise, heat flows, and thermal equilibrium becomes impossible unless the time scale of microscopic processes thermalizing this fluid is much smaller than the time scale of variation of the geometry. Sometimes this adiabatic situation occurs, for example when particle physics reactions thermalize the coupled photon-baryon fluids in the early universe [6], but rapid thermalization cannot be guaranteed to occur in general.

Here we revisit the Tolman–Ehrenfest criterion for thermal equilibrium in GR [1–3, 7] and extend it to scalar–tensor gravity. There is now a huge literature on scalar–tensor gravity, starting in the 1960s with the Jordan–Brans–Dicke theory [8] and its generalizations [9–12], continuing with the interest in string and dilaton gravity [13–15] and with a revival of scalar–tensor gravity in the past two decades. While, historically, the original motivation for Brans-Dicke gravity arose in the context of Mach’s principle [8], it was soon realized that virtually any attempt to quantum-correct GR modifies it by introducing curvature corrections to the Einstein-Hilbert action (for example, in Starobinski inflation [16] which is currently the scenario favoured by observations [17, 18]), higher order terms in the field equations, or extra degrees of freedom such as scalar fields. To wit, in Starobinski inflation quadratic corrections are added to the Ricci scalar Lagrangian of GR modifying it to $f(R)=R+\alpha R^2$. The low-energy limit of the bosonic string theory, the simplest string theory, produces an $\omega =-1$ Brans-Dicke theory (where $\omega$ is the Brans-Dicke coupling) [19, 20]. Other quadratic quantum corrections to the curvature produce fourth order field equations instead of the second order Einstein equations [21, 22].

The more recent interest in scalar–tensor gravity is motivated by the possibility of explaining the current acceleration of the universe without an *ad hoc* dark energy, mostly employing *f*(*R*) theories which are ultimately scalar–tensor ones (see [23–25] for reviews). The interest continued in the past decade with the rediscovery of Horndeski gravity [26] and its extension to degenerate higher order scalar–tensor (DHOST) theories [27–38], see [39, 39–43] for reviews.

In spite of this literature, the now old Tolman–Ehrenfest criterion for thermal equilibrium has not been extended to scalar–tensor gravity, even in the simplest scenarios. Here we fill this gap, beginning with the introduction of this criterion and its more modern derivation [44–47]. We follow the notation of Ref. [48]: *g* is the determinant of the spacetime metric $g_{ab}$, $\nabla _a$ is its covariant derivative operator, $R_{ab}$ is the Ricci tensor, while its trace $R\equiv g^{ab} R_{ab}$ is the Ricci scalar.

Consider a static spacetime endowed with a timelike Killing vector $k^a$ and a test fluid at rest in the frame adapted to this time symmetry, i.e., the fluid four-velocity is $u^a=k^a/\sqrt{ -k_ck^c}$. Fixing $u^a$ determines the $3+1$ splitting into the time direction tangent to $u^a$ and $k^a$ and the 3-space with Riemannian metric

1 $$\begin{aligned} h_{ab} = g_{ab} +u_a u_b, \end{aligned}$$

which is a purely spatial tensor according to the fluid observers, $h_{ab}u^a =h_{ab}u^b=0$. In a coordinate system $\left( t,x^i \right)$ adapted to the time symmetry, the line element reads

2 $$\begin{aligned} ds^2 = g_{00}(x^k)\, dt^2 + g_{ij}(x^k)\, d x^i\, dx^j \, \quad (i,j,k=1,2,3). \end{aligned}$$

Static observers have four-velocities parallel to the timelike Killing vector which, in these coordinates, has components $k^{\mu } = \left( k^0, 0, 0, 0 \right)$. A test fluid at rest with respect to this static observer has a position-dependent equilibrium temperature $\mathcal {T} = \mathcal {T}(x^k)$ satisfying the Tolman–Ehrenfest criterion [1–3] (see also [7, 45–47])

3 $$\begin{aligned} \mathcal {T}\sqrt{-g_{00}} = \mathcal {T}_0, \end{aligned}$$

where $\mathcal{T}_0$ is a constant. Contrary to pre-relativistic physical intuition, a fluid in thermal equilibrium in a non-uniform gravitational field necessarily has a spatial temperature gradient. This gradient expresses the fact that heat is a form of mass-energy and sinks in a gravitational field. Therefore, regions where gravity is stronger are hotter, in a way described by the gravitational shift factor $g_{00}$ [45–47]. Here thermal equilibrium is defined as the vanishing of the heat flux density $q^a = 0$.

The corresponding criterion for the equilibrium of particles with respect to diffusion in a static spacetime was formulated by Klein simply by replacing the temperature $\mathcal{T}$ with the chemical potential $\mu$ [7].

Although completely unrelated to gravity, the Tolman–Ehrenhest criterion is also of interest for thermal transport in materials. In fact, by viewing thermal transport as the linear response of a material to a temperature gradient, Luttinger described this phenomenon by introducing a counter-balancing weak gravitational field that restores thermal equilibrium in the presence of a temperature gradient [49].

The Tolman–Ehrenfest temperature is related to the Hawking temperature of a black hole seen by an observer at asymptotic infinity [50–52]. There is also interest in applying the Tolman–Ehrenfest criterion to neutron stars [53–55] and in simultaneous heat conduction and particle diffusion [56, 57], as well as in the combined equilibrium in Weyl-integrable geometries [58]. Recent work on the Tolman–Ehrenfest criterion has highlighted the severe limitations inherent in its extension to stationary but non-static geometries [45–47]. It is however, possible to extend this criterion to conformally static spacetimes, provided that the microphysics ensures that local thermal equilibrium is maintained, which is of interest in cosmology and for the Hawking radiation of dynamical black holes [59].

Back to scalar–tensor gravity: in these theories the strength of the effective gravitational coupling is $G_\textrm{eff} = 1 / \phi$, where $\phi$ is the gravitational Brans-Dicke-like scalar field. Thus, one would expect, in scalar–tensor gravity, the Tolman–Ehrenfest criterion to be modified to include $\phi$ in such a way that, in regions of space where $\phi$ is smaller (i.e., $G_\textrm{eff}$ is larger and gravity is stronger), the fluid temperature is higher. However, the Tolman–Ehrenfest criterion remains unchanged in both Jordan frame and Einstein frame scalar–tensor gravity.

To be more precise, assume that the fluid subject to our investigation is described by the dissipative stress-energy tensor

4 $$\begin{aligned} T_{ab} = \rho u_a u_b + P h_{ab} + \pi _{ab} + q_a u_b + q_b u_a\,, \end{aligned}$$

where $$\rho =T_{ab}u^a u^b $$ is the fluid energy density, $$u^a =k^a/\sqrt{-k_c k^c}$$ is its four-velocity, $$P = h_{ab} T^{ab}/3 $$ is its isotropic pressure, $$\pi _{ab}= {h_a}^c {h_b}^d T_{cd}- Ph_{ab} $$ is the anisotropic stress tensor, and $$q_a = -{h_a}^b u^c T_{bc} $$ is the heat flux density [5]. In GR, Eckart [4] generalized the phenomenological Fourier law describing heat conduction to the relativistic domain, as expressed by the constitutive relation of the fluid (4)

5 $$\begin{aligned} q_a = -\mathcal {K} h_{ab}\big (\nabla ^b\mathcal {T} + \mathcal {T}{\dot{u}}^b\big )\,, \end{aligned}$$

where $\mathcal {T}$ and $\mathcal {K}$ are the fluid temperature and thermal conductivity, respectively [5]. The only significant deviation of Eq. (5) from the non-relativistic Fourier law is the inertial term (the second term in the right-hand side brackets) contributed by the fluid’s four-acceleration $\dot{u}^a \equiv u^c\nabla _c u^a$.

It was shown by Buchdahl [60] that the four-acceleration of a particle in a static spacetime is given by

6 $$\begin{aligned} a^b \equiv \dot{u}^b = \nabla ^b\,\ln \left( \sqrt{-k^d\,k_d} \, \right) \end{aligned}$$

or, in coordinates adapted to the time symmetry (in which the line element has the form (2)),

7 $$\begin{aligned} a^b = \nabla ^b\,\ln \sqrt{-g_{00}}\,. \end{aligned}$$

Therefore, in a static spacetime and in these coordinates, the condition of thermal equilibrium $q^a=0$ for this static test fluid reads, using (5)

8 $$\begin{aligned} q_a= &   -\mathcal {K} h_{ab} \left( \nabla ^b \mathcal{T} + \mathcal {T}{\dot{u} }^b \right) \nonumber \\= &   -\mathcal{K}\mathcal{T}h_{ab}\left[ \nabla ^b \left( \ln \mathcal {T} \right) + \dot{u}^b \right] \nonumber \\= &   -\mathcal{K}\mathcal{T} h_{ab} \nabla ^b \left[ \ln \left( \mathcal {T}\sqrt{-g_{00}} \right) \right] = 0 . \end{aligned}$$

Since the projection of the gradient $\nabla ^b \left[ \ln \left( \mathcal {T}\sqrt{-g_{00}} \right) \right]$ onto the 3-space orthogonal to $u^a$ vanishes, this gradient is parallel to $u^b$, i.e., $\mathcal {T}\sqrt{-g_{00}}$ depends only on time. However, since $\mathcal{T}$ and $g_{00}$ are time-independent because of staticity, there exists an integration constant $\mathcal{T}_0$ such that Eq. (3) holds. Let us examine now scalar–tensor geometries.

## Scalar–tensor gravity

In the Jordan frame of scalar–tensor gravity, the scalar field $\phi$ couples explicitly to the Ricci scalar *R*, as described by the action [8–12]

9 $$\begin{aligned} S_\textrm{ST}= &   \frac{1}{16\pi } \int d^4x\sqrt{-g}\Big [\phi R - \frac{\omega (\phi )}{\phi }\,\nabla ^a\phi \nabla _a\phi -V(\phi )\Big ] \nonumber \\  &   + S_\mathrm {(m)} = S_\mathrm {(grav)}+ S_\mathrm {(m)}, \end{aligned}$$

where $\omega (\phi )$ is the Brans–Dicke coupling, $V(\phi )$ is a potential for the Brans–Dicke field $\phi$, while $S_\mathrm {(m)}$ is the matter part of the action. Varying the Jordan frame action (9) with respect to $g^{ab}$ yields the field equations

10 $$\begin{aligned} G_{ab}= \frac{8\pi }{\phi } \, T^\mathrm {(m)}_{ab} +T^{(\phi )}_{ab}, \end{aligned}$$

which can be read as effective Einstein equations where $T^\mathrm {(m)}_{ab}$ is the matter stress-energy tensor and

11 $$\begin{aligned} T^{(\phi )}_{ab}= &   \frac{\omega }{\phi ^2} \left( \nabla _a \phi \nabla _b \phi -\frac{1}{2} \, g_{ab} \nabla _c \phi \nabla ^c \phi \right) \nonumber \\  &   +\frac{1}{\phi } \left( \nabla _a \nabla _b \phi - g_{ab} \Box \phi \right) -\frac{V}{2\phi }\, g_{ab} \end{aligned}$$

is an effective stress-energy tensor of the form (4) [61]. The variation of the action (9) with respect to $\phi$ gives

12 $$\begin{aligned} \Box \phi = \frac{1}{2\omega +3} \left( \frac{8\pi T^{(m)} }{\phi } + \phi \, \frac{d V}{d\phi } -2V -\frac{d\omega }{d\phi } \, \nabla ^c \phi \nabla _c \phi \right) \,, \end{aligned}$$

where $\Box \equiv g^{ab} \nabla _a \nabla _b$ is the curved space d’Alembertian and $T^\mathrm {( m)} \equiv g^{ab} T^\mathrm {( m)}_{ab}$.

The conformal transformation

13 $$\begin{aligned} g_{ab} \rightarrow \tilde{g}_{ab} = \Omega ^2\,g_{ab} = \phi \,g_{ab}\, \end{aligned}$$

and the scalar field redefinition $\phi \rightarrow \tilde{\phi }$ given in differential form by

14 $$\begin{aligned} d\tilde{\phi } = \sqrt{ \frac{2\omega +3}{16\pi } } \, \frac{d\phi }{\phi } \end{aligned}$$

bring the Jordan frame action (9) into its Einstein frame version, in which the gravitational sector of the theory looks like the Einstein–Hilbert action

15 $$\begin{aligned} S_\mathrm {(grav)} = \int d^4x \sqrt{-\tilde{g}}\left[ \frac{\tilde{R}}{16\pi } - \frac{1}{2} \,\tilde{g}^{ab} \tilde{\nabla }_a \tilde{\phi } \tilde{\nabla _b} \tilde{\phi } -U \left( \tilde{\phi } \right) \right] \end{aligned}$$

where a tilde denotes quantities constructed with $\tilde{g}_{ab}$, and

16 $$\begin{aligned} U \left( \tilde{\phi } \right) = \frac{ V \left[ \phi \left( \tilde{\phi } \right) \right] }{ \phi ^2 \left( \tilde{\phi } \right) }\,. \end{aligned}$$

In the Einstein frame, the equation of timelike geodesics is modified to [62]

17 $$\begin{aligned} \tilde{ \dot{u}}^a \equiv \tilde{u}^b \tilde{\nabla }_b \tilde{u}^a = \sqrt{ \frac{4\pi }{2\omega +3} } \, \tilde{\nabla }^a \tilde{\phi } \,. \end{aligned}$$

Using $\tilde{\nabla }_a \tilde{\phi } =\sqrt{ \frac{2\omega +3}{16\pi }} \, \nabla _a \ln \phi$, this corrected geodesic equation reads

18 $$\begin{aligned} \tilde{ \dot{u}}^a = \tilde{\nabla }^a \left( \ln \sqrt{\phi } \right) \,. \end{aligned}$$

Massive test particles subject only to gravity feel both the metric and the gravitational scalar field $\phi$ (or $\tilde{\phi }$) and deviate from geodesics.

The decomposition (4) of the stress-energy tensor applies to any symmetric two-index tensor [63], thus the stress-energy tensor $\tilde{T}_{ab}$ of the Einstein frame fluid can be decomposed as

19 $$\begin{aligned} \tilde{T}_{ab} = \tilde{\rho } \, \tilde{u}_a \tilde{u}_b + \tilde{P} \tilde{h}_{ab} + \tilde{\pi }_{ab} + \tilde{q}_a \tilde{u}_b + \tilde{q}_b \tilde{u}_a\,, \end{aligned}$$

without extra assumptions on the conformal fluid quantities $\tilde{\rho }$, $\tilde{P}$, $\tilde{\pi }_{ab}$ and $\tilde{q}_a$.

The physical implications of conformal transformations, including the scaling of dimensional physical quantities and of their units were discussed long ago by Dicke [62]. According to [62], only the ratio of a dimensional quantity to its unit (that scales the same way with powers of $\Omega$) is physically measurable, therefore physics in the rescaled geometry $\tilde{g}_{ab}$ is equivalent to the same physics in the non-rescaled geometry $g_{ab}$, provided that ratios of quantities are considered instead of the quantities themselves. This is easier said than done and Dicke’s point of view, in particular with regard to the physical equivalence of Jordan and Einstein frames, has been the subject of a heated debate that continues to these days (see [64] for a review). However, it is hard to argue with the fact that, when measuring dimensional quantities one measures only a ratio of a quantity to its unit. The complications and the disagreements arise when derivatives of these quantities, or their combinations, come into play. However, to discuss the Tolman–Ehrenfest criterion one needs only the temperature, which is an exceptionally simple situation and no such complication is involved.

Dicke showed that, under the conformal scaling (13), masses scale as $\tilde{m} = \Omega ^{-1}\,m$ while times and lengths scale as $\Delta \tilde{\ell } \sim \Delta \tilde{t} \sim \Omega$. Since the speed of light *in vacuo* *c* is a ratio of space and time, it is conformally invariant, and since energy has dimensions $[Mc^2]$, it scales like a mass [62, 64]. The product $k_B \mathcal{T}$ (where $k_B$ is the Boltzmann constant) is dimensionally a mass and scales as $k_B \tilde{\mathcal{T}}\sim \Omega ^{-1}$. Since the Boltzmann constant $k_B$ is a true constant, then $\tilde{\mathcal{T}} \sim \Omega ^{-1}$ (which is in agreement with our Eq. (25) below).

## Thermal equilibrium in scalar–tensor gravity

As in Einstein gravity, thermal equilibrium of a static test fluid in a static spacetime is defined as the absence of heat fluxes, $q^a=0$. We discuss the Jordan frame and the Einstein frame and show how the two descriptions give the same result.

### Jordan frame

In the Jordan frame, assume:

- a static spacetime with line element (2) in coordinates adapted to the time symmetry, in which the timelike Killing vector defines the time direction, $k^a=\left( \partial /\partial t \right) ^a$;
- a static scalar field $\phi =\phi \left( x^i \right)$;
- a test fluid described by the stress-energy tensor (4), with four-velocity parallel to the timelike Killing vector $k^a$ and satisfying the Eckart constitutive relation (5). This fluid is assumed to be at rest in the static frame.

Then, any observer comoving with this fluid will see no heat flow, $q^a=0$, and necessarily thermal equilibrium.

The assumption that the Jordan frame fluid satisfies the Eckart constitutive relation is crucial, but its legitimacy is not clear a priori. While Eckart’s relation seems natural since it expresses the response of the fluid to temperature gradients, the fluid is now also in thermal equilibrium with the scalar field $\phi$, thus things could change. We will justify this assumption in the next subsection by mapping a more familiar Einstein frame result back to the Jordan frame and showing that Eckart’s relation holds in the Jordan frame if and only if it holds in the Einstein frame. With this *caveat*, let’s proceed. The derivation of the Tolman–Ehrenfest criterion $T\sqrt{-g_{00}}=$ const. then proceeds the same way as in GR.

### Einstein frame

We now derive the Tolman–Ehrenfest criterion in the Einstein conformal frame, where the theory formally looks like GR and then we use it to justify the Eckart constitutive relation and the Tolman–Ehrenfest criterion in Jordan frame scalar–tensor gravity.

The key idea of the calculation is that, since we know the criterion for thermal equilibrium in GR, and that Einstein frame gravity formally looks like GR, we can begin our discussion in this conformal frame and then map the Tolman criterion back to the Jordan frame, which is the one most commonly used to formulate physics in scalar–tensor gravity. As explained in Appendix A, while the Einstein frame fluid couples explicitly to the scalar $\tilde{\phi }$, this coupling is automatically included in Eckart’s constitutive relation and does not need to be supplemented by extra heat flux density terms introduced by hand.

Since $\Omega =\sqrt{\phi \left( x^i\right) }$ is static, $u^b\nabla _b\Omega = 0$, and the conformal geometry with metric $\tilde{g}_{ab}=\phi \, g_{ab}$ is also static. An exception is given by stealth solutions of the scalar–tensor field equations in which $g_{ab}= \eta _{ab}$ (the Minkowski metric), $\phi$ does not gravitate, but it is non-trivial and time-dependent [65–71]. This situation is discussed in Sect. 4.

Since $\tilde{g}_{ab}=\Omega ^2 \, g_{ab}$ is static, the Buchdahl relation [60] holds in the rescaled world,

20 $$\begin{aligned} \tilde{a}^b = \tilde{\nabla }^b\left( \ln \sqrt{-\tilde{g}_{00}} \right) \,. \end{aligned}$$

This relation can also be proved directly in the Einstein frame (see Appendix A).

Under a conformal transformation $\tilde{T}_{ab}$ maintains its dissipative fluid form (19), which is common to any symmetric two-index tensor regardless of its nature [63]. Furthermore, the $\big (3+1\big )$ splitting of spacetime into space and time does not change because the time direction $\tilde{u}^c = u^c / \Omega$ of the observers comoving with the fluid in the tilded world is parallel to the time direction $u^c$ of the observers comoving with the Jordan frame fluid, while $\tilde{h}_{ab}=\Omega ^2 \, h_{ab}$ is just a rescaling of the Riemannian 3-metric $h_{ab}$. Then, $g_{ab} = -u_au_b + h_{ab}$ has the parallel decomposition

21 $$\begin{aligned} \tilde{g}_{ab} = \Omega ^2g_{ab} = \Omega ^2\big (-u_au_b + h_{ab}\big )=-\tilde{u}_a\tilde{u}_b + \tilde{h}_{ab} \end{aligned}$$

in the tilded world, as follows from the fact that $\tilde{u}_a = \Omega \, u_a$ and $\tilde{h}_{ab}=\Omega ^2 \, h_{ab}$. Since $h_{ab}u^a = h_{ab}u^b = 0$, it is also $\tilde{h}_{ab} \tilde{u}^a = \tilde{h}_{ab} \tilde{u}^b = 0$ and purely spatial vectors or tensors with respect to $g_{ab}$ remain purely spatial with respect to $\tilde{g}_{ab}$. In particular the Einstein frame counterpart of the heat flux density, which satisfies $\tilde{q}^c =0$, is purely spatial, i.e., $\tilde{q}_c \tilde{u}^c = 0$.

As in the Jordan frame, in the Einstein conformal frame the spacetime is static and the test fluid is at rest in the static frame of the metric $\tilde{g}_{ab}$. Thermal equilibrium is defined again as the absence of heat flux, i.e., $\tilde{q}_a = 0$. If one is willing to assume that the constitutive relation (5) is valid in the Jordan frame, it should also hold for the conformal fluid in the Einstein frame. Indeed, under a conformal transformation $g_{ab}\rightarrow \Omega ^2 g_{ab}$, the heat flux density transforms according to $\tilde{q}_a=\Omega ^{-3} q_a$ (see Eq. (36) below). As a consequence, the notion of thermal equilibrium does not depend on the conformal frame: $q^a$ vanishes in the Jordan frame if and only if $\tilde{q}^a$ vanishes in the Einstein frame. Indeed, it would be worrisome if thermal equilibrium depended on the conformal frame.

Then, at thermal equilibrium, it must be

22 $$\begin{aligned} \tilde{q}_a= &   -\tilde{\mathcal {K}}\tilde{h}_{ab}\left( \tilde{\nabla }^b \tilde{\mathcal {T}} + \tilde{\mathcal {T}}{\dot{\tilde{u}}}^b \right) \nonumber \\= &   -\tilde{\mathcal {K}}\tilde{\mathcal {T}}\tilde{h}_{ab}\left[ \tilde{\nabla }^b\big (\ln \tilde{\mathcal {T}}\big ) + \dot{\tilde{u}}^b \right] \nonumber \\= &   -\tilde{\mathcal {K}}\tilde{\mathcal {T}}\tilde{h}_{ab} \tilde{\nabla }^b \left[ \ln \left( \tilde{\mathcal {T}}\sqrt{-\tilde{g}_{00}} \right) \right] = 0, \end{aligned}$$

where Eq. (20) was used.

The Einstein frame fluid being at thermal equilibrium implies that $\tilde{\nabla }^b \left[ \ln \left( \tilde{\mathcal {T}}\sqrt{-\tilde{g}_{00}}\right) \right]$ is parallel to $\tilde{u}^b$ and orthogonal to $\tilde{h}_{ab}$, there exists an integration constant $\tilde{\mathcal {T}}_0$ such that

23 $$\begin{aligned} \tilde{\mathcal {T}}\sqrt{-\tilde{g}_{00}} = \tilde{\mathcal {T}}_0\,, \end{aligned}$$

a near identical match with the Tolman–Ehrenfest criterion (3) of the Jordan frame. Dividing term to term Eqs. (23) and (3) gives

24 $$\begin{aligned} \tilde{\mathcal {T}}\Omega \sqrt{-g_{00}} = \bigg (\frac{\tilde{\mathcal {T}}_0}{ \mathcal {T}_0}\bigg )\,\mathcal {T}\sqrt{-g_{00}} \end{aligned}$$

and, absorbing the ratio of the constants $\mathcal {T}_0$ and $\tilde{\mathcal {T}}_0$ into $\Omega$ via a coordinate redefinition, this simplifies to

25 $$\begin{aligned} \tilde{\mathcal {T}} = \frac{ \mathcal {T} }{\Omega } \,. \end{aligned}$$

This relation has been demonstrated for more general conformally static spacetimes in the context of GR [59]. The most physical example is the scaling of the cosmic microwave background with the scale factor of the Friedmann–Lemaître–Robertson–Walker (FLRW) universe in which it lives [59]. Restrict, for simplicity, to a spatially flat FLRW universe with line element

26 $$\begin{aligned} ds^2=- d t^2 + a^2(t)\left( dx^2+ dy^2+ dz^2 \right) \end{aligned}$$

in comoving coordinates $\big (t,x,y,z\big )$. The use of the conformal time $\eta$, defined by $dt = a \, d\eta$, makes the conformal flatness of this geometry explicit:

27 $$\begin{aligned} ds^2=a^2(\eta )\big (- d\eta ^2 + dx^2+dy^2+dz^2\big )\,, \end{aligned}$$

with conformal factor $\Omega$ equal to the scale factor $a(\eta )$. Long after equipartition, the cosmic microwave background becomes a test fluid in local thermal equilibrium that evolves decoupled from other forms of matter. To maintain the Planck spectral energy density distribution

28 $$\begin{aligned} u\left( \nu , \mathcal{T} \right) = \frac{8\pi h\nu ^3}{c^3} \, \frac{1}{\text{ e}^{\frac{h\nu }{k_B \mathcal{T} }} -1} \end{aligned}$$

(where *h* is the Planck constant, $k_B$ is the Boltzmann constant, $\nu$ is the photon frequency, and we restore the speed of light *c*), the temperature $\mathcal{T}$ of the cosmic microwave background must scale as $\mathcal{T} \propto 1/a$ because the proper wavelength $\lambda$ scales with the scale factor *a* like all physical lengths, i.e., $h\nu = hc / \lambda \propto 1/a$. The scaling $\mathcal{T} \propto 1/a$ is nothing but the scaling $\tilde{ \mathcal{T}} = \mathcal{T}/ \Omega$ derived here, but in a very different context. Here we discuss thermal equilibrium (restricted to static spacetimes) in the context of scalar–tensor gravity, whereas Ref. [59] treats conformally static time-dependent spacetimes in GR.

The transformation of the four-acceleration $\tilde{\dot{u}}^a$ under a conformal transformation $g_{ab} \rightarrow \tilde{g}_{ab} = \Omega ^2 g_{ab}$,

29 $$\begin{aligned} \tilde{\dot{u}}^a = \frac{\dot{u}^a }{\Omega ^2} +\frac{ \nabla ^a \Omega }{\Omega ^3} + u^a \left( \frac{u^c \nabla _c \Omega }{\Omega } \right) \,, \end{aligned}$$

is derived in Appendix A.

Let us examine the transformation properties of the stress-energy tensor of the dissipative test fluid. The variational definition of the Einstein frame stress-energy tensor

30 $$\begin{aligned} \tilde{T}_{ab}= &   \frac{-2}{ \sqrt{ -\tilde{g} } } \, \frac{ \delta \left( \sqrt{ -\tilde{g} } \, \tilde{ \mathcal{L}}^\mathrm {(m)} \right) }{\delta \tilde{g}^{ab} }, \end{aligned}$$

where $\tilde{ \mathcal{L}}^\mathrm {(m)}$ is the matter Lagrangian density in the Einstein frame, implies that $\tilde{ \mathcal{L}}^\mathrm {(m)}=\Omega ^{-4} \mathcal{L}^\mathrm {(m)}$, and $\sqrt{-\tilde{g}} \, \tilde{ \mathcal{L}}^\mathrm {(m)}= \sqrt{-g} \, \mathcal{L}^\mathrm {(m)}$, and (e.g., [72])

31 $$\begin{aligned} \tilde{T}^{ab} = \Omega ^{-6}\,T^{ab}, \quad \tilde{T}_{ab} = \Omega ^{-2} \,T_{ab}\,, \end{aligned}$$

Using this fact and $\tilde{u}_a = \Omega u_a$, $\tilde{h}_{ab}=\Omega ^2 h_{ab}$, one writes

32 $$\begin{aligned} \tilde{T}_{ab}= &   \frac{T_{ab}}{\Omega ^2} = \frac{\rho u_a u_b}{\Omega ^2} + \frac{ P h_{ab}}{\Omega ^2} + \frac{ \pi _{ab}}{\Omega ^2} + q_a \, \frac{ u_b}{\Omega ^2} + q_b \, \frac{ u_a}{\Omega ^2} \nonumber \\= &   \frac{\rho }{\Omega ^2} \, \frac{\tilde{u}_a}{\Omega } \, \frac{\tilde{u}_b}{\Omega } + \frac{ P \tilde{h}_{ab}}{\Omega ^4} + \frac{ \pi _{ab}}{\Omega ^2} + \frac{ q_a}{\Omega ^2} \, \frac{ \tilde{u}_b}{\Omega } + \frac{ q_b}{\Omega ^2} \, \frac{ \tilde{u}_a}{\Omega } \nonumber \\= &   \tilde{\rho } \tilde{u}_a \tilde{u}_b + \tilde{P} \tilde{h}_{ab} + \tilde{\pi }_{ab} + \tilde{q}_a \tilde{u}_b + \tilde{q}_b \tilde{u}_a , \end{aligned}$$

where

33 $$\begin{aligned} \tilde{\rho }= &   \Omega ^{-4} \rho , \end{aligned}$$

34 $$\begin{aligned} \tilde{P}= &   \Omega ^{-4} P , \end{aligned}$$

35 $$\begin{aligned} \tilde{\pi }_{ab}= &   \Omega ^{-2} \pi _{ab} , \end{aligned}$$

36 $$\begin{aligned} \tilde{q}_a= &   \Omega ^{-3} q_a . \end{aligned}$$

The transformation properties of $\rho$ and *P* for perfect and imperfect fluids are well known in the literature [59, 72]. The scaling of the heat flux density $\tilde{q}_a=\Omega ^{-3} q_a$ shows that thermal equilibrium in the Jordan frame (i.e., $q_a=0$) is equivalent to thermal equilibrium in the Einstein frame ($\tilde{q}_a=0$). All these rescalings are consistent with Dicke’s [62] dimensional arguments.

Let us show now that the Eckart constitutive relation in the Jordan frame follows from that in the Einstein frame. Using the transformation properties $\tilde{ \mathcal{T}} = \mathcal{T}/\Omega$, $\tilde{q}_a = \Omega ^{-3} q_a$ and Eq. (29), the Einstein frame relation

37 $$\begin{aligned} \tilde{q}_a = -\tilde{K} \tilde{h}_{ab} \left( \tilde{\nabla }^b \tilde{ \mathcal {T} } + \tilde{ \mathcal {T} } \tilde{\dot{u} }^b \right) \end{aligned}$$

yields

38 $$\begin{aligned} \Omega ^{-3} q_a= &   -\tilde{K} \Omega ^2 h_{ab} \left[ \Omega ^{-2} g^{bc} \nabla _c \left( \frac{ \mathcal {T} }{\Omega } \right) \right. \nonumber \\  &   \left. + \frac{ \mathcal {T} }{\Omega } \left( \frac{ \dot{u}^b }{ \Omega ^2} +\frac{\nabla ^b \Omega }{\Omega ^3} + \frac{u^c \nabla _c\Omega }{\Omega ^3} \right) \right] \nonumber \\= &   -\tilde{K} \Omega ^2 h_{ab} \left( \frac{\nabla ^b \mathcal{T} }{\Omega ^3} -\frac{ \mathcal{T} \nabla ^b \Omega }{\Omega ^4} + \frac{ \mathcal{T} \dot{u}^b }{\Omega ^3} + \frac{ \mathcal{T} \nabla ^b \Omega }{\Omega ^4} \right) \nonumber \\= &   - \frac{ \tilde{K} \Omega ^2 }{\Omega ^3} \, h_{ab} \left( \nabla ^b \mathcal{T} +\mathcal{T} \dot{u}^b \right) , \end{aligned}$$

which reproduces the Eckart generalization of Fourier’s law with $\tilde{K}=\Omega ^{-2} K$. By repeating these steps in reverse, one obtains Eckart’s law in the Einstein frame from that in the Jordan frame. Therefore, Eckart’s law holds in the Einstein frame if and only if it holds in the Jordan frame.

## Stealth solutions

Let us consider now stealth solutions of the form $g_{ab}=\eta _{ab}$, $\phi =\phi (t)$ (where $\eta _{ab}$ is the Minkowski metric). These solutions [65–71] are not possible in GR and are typical of scalar–tensor gravity. For these exotic stealth solutions, the effective stress-energy tensor $T_{ab}^{(\phi )}$ of the scalar field vanishes due to cancellations between its terms. Therefore, the Brans–Dicke-like scalar $\phi$ does not gravitate, but the effective gravitational coupling $G_\textrm{eff}=1/\phi$ felt by test matter changes in time.

The derivation of the criterion of thermal equilibrium in GR and in scalar–tensor gravity becomes invalid because, for stealth solutions, the Einstein frame metric $\tilde{g}_{ab}=\phi (t) \eta _{ab}$ is now time-dependent and the Buchdahl relation (6) does not apply. However, the Einstein frame metric $\tilde{g}_{ab}$ is special since it is conformally flat, and the discussion of Ref. [59] applies to such situations. We do not repeat that discussion here, but report the salient logical steps. Although the Buchdahl relation is invalid, in conformally flat geometries^1^$\tilde{g}_{ab}=\Omega ^2(t) \eta _{ab}$, its projection onto the 3-space orthogonal to the fluid four-velocity $\tilde{u}^a$ still holds, that is,

39 $$\begin{aligned} \tilde{h}_{ab} \tilde{a}^b = \tilde{h}_{ab} \tilde{\nabla }^b \ln \sqrt{-\tilde{g}_{00} } \,. \end{aligned}$$

This fact is sufficient to conclude again that $\tilde{ \mathcal{T}}=\mathcal{T}/\Omega$. Examples of this relation are the scaling of the temperature of the cosmic microwave background $\mathcal{T}\sim 1/a$ with the scale factor of the FLRW universe discussed in Sect. 3, and the scaling of the Hawking temperature of dynamical black holes embedded in a FLRW universe [59]. Hence, for stealth solutions, the Jordan frame result is still valid and $\mathcal{T}$ does not depend on time. Observers comoving with the (Jordan frame) test fluid would find themselves in Minkowski space and would observe the fluid in thermal equilibrium with no heat flux and a temperature uniform and constant in time. Stealth solutions of the field equations of scalar–tensor gravity are undoubtedly rather exotic and we will not discuss them further.

## Conclusions

We have extended the Tolman–Ehrenfest criterion for thermal equilibrium in GR to scalar–tensor gravity. The valid GR criterion (3) also holds in Jordan frame and Einstein frame scalar–tensor gravity. One would expect the strength of the gravitational coupling $G_\textrm{eff}=1/\phi$ to affect thermal equilibrium, but this is not the case. This fact is surprising because the essence of thermal equilibrium in GR (expressed by the Tolman criterion (3)) is that heat is a form of mass-energy, therefore it sinks in a gravitational field. While, in GR, the gravitational coupling strength *G* is a true constant, in scalar–tensor gravity it is not, thus one expects heat to sink more where the gravitational coupling strength $G_\textrm{eff}$ is higher and $\phi$ to appear in the condition for thermal equilibrium in the Jordan frame of scalar–tensor gravity. However, neither the field equations nor the conservation of the fluid’s stress-energy tensor (which fails in the Einstein frame [62]) are used in the derivation of the Tolman–Ehrenfest criterion.

It is now tempting to consider extending the Tolman–Ehrenfest criterion to Horndeski and DHOST gravity, but the transformation to a sort of analogous Einstein frame becomes much more complicated [27–39, 39–43]. It is a disformal transformation involving first (and possibly higher order [73, 74]) derivatives of the scalar field, which transforms the theory to a much more complicated and analogous Einstein frame where the role of scaling units (or its generalization) is completely unclear. Since the field equations are not used, however, one expects the Tolman–Ehrenfest criterion to hold in any theory of gravity. The result presented here will be useful in studies of the cosmic microwave background and of Hawking radiation in scalar–tensor gravity, in the same way that the Tolman criterion is applied to these subjects in GR [59, 75].

## Data Availability

Due to the theoretical nature of this work, no data have been used.

## Appendix A: Transformation of the four-acceleration and Buchdahl relation in the Einstein frame

Here we derive the transformation of the four-acceleration and we prove the Buchdahl relation directly in the Einstein frame using the transformation properties of kinematic and geometric quantities under conformal rescalings. In fact, the four-velocities normalizations $g^{ab} u_a u_b=-1$ and $\tilde{g}^{ab} \tilde{u}_a \tilde{u}_b=-1$ imply that

A1 $$\begin{aligned} \tilde{u}^a = \frac{ u^a}{\Omega } \,,\quad \quad \tilde{u}_a = \Omega \, u_a\,. \end{aligned}$$

The four-acceleration $\tilde{a}^b$ in the tilded world is calculated in terms of the four-acceleration $a^b$, obtaining

A2 $$\begin{aligned} \tilde{\dot{u}}^b\equiv &   \tilde{u}^c \tilde{\nabla }_c \tilde{u}^b = \frac{ u^c}{\Omega } \, \tilde{\nabla }_c \left( \frac{ u^b}{\Omega } \right) \nonumber \\= &   \frac{u^c}{\Omega ^2} \left( \partial _c u^b +\tilde{\Gamma }^b_{cd} u^d \right) -u^b \left( \frac{ u^c\nabla _c \Omega }{\Omega ^3} \right) \nonumber \\= &   \frac{u^c}{\Omega ^2} \left\{ \partial _c u^b + \left[ \Gamma ^b_{cd} + \frac{1}{\Omega } \left( {\delta ^b}_c \nabla _d \Omega + {\delta ^b}_d \nabla _c\Omega - g_{dc} \nabla ^b \Omega \right) \right] u^d \right\} \nonumber \\  &   -u^b \left( \frac{ u^c\nabla _c \Omega }{\Omega ^3} \right) \nonumber \\= &   \frac{u^c}{\Omega ^2} \left[ \left( \partial _c u^b + \Gamma ^b_{cd}u^d \right) + \frac{1}{\Omega } \left( {\delta ^b}_c \nabla _d \Omega + u^b \nabla _c\Omega - u_c \nabla ^b \Omega \right) \right] \nonumber \\  &   -u^b \left( \frac{ u^c\nabla _c \Omega }{\Omega ^3} \right) \nonumber \\= &   \frac{1}{\Omega ^2} \left[ u^c \nabla _c u^b + \frac{1}{\Omega } \left( u^b u^d \nabla _d \Omega + u^b u^c \nabla _c\Omega + \nabla ^b \Omega \right) \right. \nonumber \\  &   \left. -u^b \left( \frac{ u^c\nabla _c \Omega }{\Omega } \right) \right] \nonumber \\= &   \frac{ \dot{u}^c}{\Omega ^2} + \frac{\nabla ^b \Omega }{\Omega ^3 } + u^b \left( \frac{u^c \nabla _c \Omega }{\Omega ^3} \right) . \end{aligned}$$

As a check, in the Einstein frame test particles subject only to gravity (which follow geodesics of the Jordan frame, $\dot{u}^a=0$) do not follow geodesics of the metric $\tilde{g}_{ab}$ but are subject to the acceleration $\frac{\nabla ^b \Omega }{\Omega ^3 } + u^b \left( \frac{u^c \nabla _c \Omega }{\Omega ^3} \right)$. The second term is parallel to the four-velocity $u^b$ and can be removed by going to an affine parametrization, but there remains the residual four-acceleration

A3 $$\begin{aligned} \frac{\nabla ^b \Omega }{\Omega ^3 }= \frac{\nabla ^b \phi }{2 \phi ^2 }= \frac{ \tilde{\nabla }^b \phi }{2 \phi }= \tilde{\nabla }^b \left( \ln \sqrt{\phi } \right) \,, \end{aligned}$$

which reproduces Eq. (18). It is important to note that the explicit coupling of the fluid to $\tilde{\phi }$ (or to $\phi$) is already taken into account in the fluid’s four-acceleration and does not need to be inserted by hand in the Einstein frame Eckart relation $\tilde{q}_a = - \tilde{K}\tilde{h}_{ab} \left( \tilde{\nabla }^b \tilde{ \mathcal{T} } +\tilde{ \mathcal{T} } \tilde{\dot{u} }^b \right)$. In other words, one does not need to add another heat flux term depending on $\tilde{\nabla }_a \phi$ in this relation. The physical interpretation of this fact is that, although the fluid is coupled to $\phi$ (in fact, because of this coupling), it is always in thermal equilibrium with it and there is no heat flow between this fluid and $\phi$.

Now $\tilde{a}^b$ can be rewritten by using $a^b$ given by Eq. (7), which yields^2^

A4 $$\begin{aligned} \tilde{a}^b= &   \dot{\tilde{u}}^b = \Omega ^{-2}\bigg [ \nabla ^b\,\Big (\ln \sqrt{-g_{00}}\Big ) + \frac{\nabla ^b\,\Omega }{\Omega }\bigg ] \nonumber \\= &   \tilde{\nabla }^b\,\Big ( \ln \sqrt{-g_{00}} + \ln \Omega \Big ) = \tilde{\nabla }^b \left( \ln \sqrt{-\tilde{g}_{00}} \right) , \end{aligned}$$

where we used the fact that

A5 $$\begin{aligned} \tilde{\nabla }^bf = \tilde{g}^{bc}\,\tilde{\nabla }_cf = \Omega ^{-2}\,g^{bc}\,\nabla _cf = \Omega ^{-2}\nabla ^b f \end{aligned}$$

for any scalar function *f*.

## Footnotes

1 The discussion of Ref. [59] is more general since it applies to *conformally static* geometries.
